# The dual amylin and calcitonin receptor agonist KBP-336 elicits a unique combination of weight loss, antinociception and bone protection – a novel disease-modifying osteoarthritis drug

**DOI:** 10.1186/s13075-024-03361-2

**Published:** 2024-07-12

**Authors:** Khaled Elhady Mohamed, Anna Thorsø Larsen, Simone Melander, Frederik Andersen, Ellen Barendorff Kerrn, Morten Asser Karsdal, Kim Henriksen

**Affiliations:** 1 Nordic Bioscience Biomarkers and Research, Herlev Hovedgade 207, Herlev, DK-2730 Denmark; 2 KeyBioscience AG, Stans, Switzerland; 3 Department of Drug Design and Pharmacology, University of Copenhagen, Copenhagen, Denmark; 4 Department of Molecular and Medical Biology, Roskilde University Center, Roskilde, Denmark

**Keywords:** Obesity, Osteoarthritis, Pain, DACRA, Calcitonin, Amylin

## Abstract

**Background:**

Despite the extensive research to provide a disease-modifying osteoarthritis drug (DMOAD), there is still no approved DMOAD. Dual amylin and calcitonin receptor agonists (DACRA) can provide metabolic benefits along with antinociceptive and potential structural preserving effects. In these studies, we tested a DACRA named KBP-336 on a metabolic model of OA in meniscectomised (MNX) rats.

**Methods:**

We evaluated KBP-336’s effect on pain-like symptoms in Sprague Dawley (SD) rats on high-fat diet (HFD) that underwent meniscectomy using the von Frey test to measure the 50% paw withdrawal threshold (PWT) and analyzed using one-way ANOVA. Short in vivo studies and in vitro cell receptor expression systems were used to illustrate receptor pharmacology.

**Results:**

After 30 weeks on HFD, including an 8-week treatment, female MNX animals receiving KBP-336 4.5 nmol/Kg/72 h had lower body weight and smaller adipose tissues than their vehicle-treated counterparts. After 20 weeks on HFD, including an 8-week treatment, male rats receiving KBP-336 had lower body weight than the vehicle group. In both the female and male rats, the MNX groups on KBP-336 treatment had a higher PWT than the vehicle-treated MNX group. Aiming to identify the receptor influencing pain alleviation, KBP-336 was compared to the long-acting human calcitonin (hCTA). Single-dose studies on 12-week-old male rats showed that hCTA lowers CTX-I without affecting food intake, confirming its calcitonin receptor selectivity. On the metabolic OA model with 18 weeks of HFD, including 6-week treatment, hCTA at 100 nmol/Kg/24 h and KBP-336 at 0.5, 1.5, and 4.5 nmol/Kg/72 h produced significantly higher PWT in MNX animals compared to MNX animals on vehicle treatment. hCTA and KBP-336 at 0.5 nmol/Kg did not affect body weight and fat tissues.

**Conclusion:**

Overall, KBP-336 improved the pain observed in the metabolic OA model. Calcitonin receptor activation proved to be essential in this antinociceptive effect.

**Supplementary Information:**

The online version contains supplementary material available at 10.1186/s13075-024-03361-2.

## Introduction

Osteoarthritis (OA) has been one of the significant burdens on healthcare systems globally, with over 595 million cases in 2020 [1]. OA affects articular joints like hands, hips, and knees, with knee OA responsible for more than half the total OA cases [1]. Patients suffer from pain, cramps, stiffness, reduced range of motion, and restricted physical functionality, severely affecting their quality of life [2]. Multiple factors are involved in the etiology of OA, revealing a complex disease affecting a heterogeneous patient population [3]. However, the surging obesity pandemic is one of the main drivers of OA [4]. In addition to the increased mechanical direct stress on the knee and hip joints, obesity harms articular joints in other mechanisms, as surveys show an elevated incidence of hand OA in obese individuals [5]. Although OA societies’ guidelines recommend weight loss as a core treatment for all overweight and obese OA patients, weight loss pharmacotherapies remain an untapped potential treatment of OA [6].

OA pain worsens with increased obesity, as visceral adiposity, higher BMI, and increased waist circumference correlate with increased OA pain [7, 8]. The severity of OA pain increased in a dose-dependent manner with higher BMI [9]. The aggravation of OA pain by obesity can be through increased mechanical loading [10], and elevated proinflammatory cytokines released from adipose tissues [11]. On the other hand, weight loss can improve the pain experienced by OA patients. A 10% or more decrease in weight proved to be sufficient to decrease OA pain in patients with obesity [12, 13]. The Intensive Diet and Exercise for Arthritis (IDEA) clinical trial concluded that diet and exercise were effective in enhancing mobility and decreasing WOMAC pain and function scores [14].

Currently, there is still no approved disease-modifying osteoarthritis drug (DMOAD) [6, 15]. Many past clinical failures did not relieve pain or avoid total joint replacement surgery, as with Sprifermin and Tanezumab, respectively [16–18]. Although obesity plays a considerable role in structural and symptomatic deterioration in OA, until lately, only one RCT tested an anti-obesity drug on OA [19]. But recently, the glucagon-like peptide 1 receptor agonist (GLP-1) semaglutide has been shown to be effective in significantly improving WOMAC scores in OA patients with BMI > 30, opening the door for more antiobesity drugs as a potential treatment for OA [20]. Additionally, adding weight loss benefits to structural preservation and pain-relieving properties can provide attractive DMOAD candidates.

Dual amylin and calcitonin receptor agonists (DACRAs) are an exciting class of peptides that can potentially provide a DMOAD. DACRAs like KeyBioscience Peptides (KBPs) have shown potent metabolic properties. KBPs have been derived from salmon calcitonin (sCT), a DACRA shown to activate both the calcitonin and the amylin receptors [21]. Namely, KBP-336 has shown selectivity to the calcitonin and the amylin receptors while exhibiting a lack of activation of other members of the calcitonin family, such as the calcitonin gene-related peptide and adrenomedullin receptors; this lack of off-target activity is critical, as agonism of the CGRP-R or the AM-R likely would introduce undesirable effects such as vasodilation [22, 23]. KBPs’ activation of the amylin receptor provided decreased food intake and body weight and reduced the size of adipose tissues in preclinical models of obesity. Furthermore, KBPs reduced blood glucose levels and improved insulin sensitivity in diabetic rat models [24–26]. The satiety effects of DACRAs are known to be mediated through the activation of amylin receptors in the area postrema [27]. On the other hand, DACRAs act as an antiresorptive on bones through the disruption of osteoclastic resorption by calcitonin receptor activation [28]. Moreover, DACRAs provide pain relief, which is an essential property of a DMOAD. sCT has been used for bone-related pain, especially osteoporosis fracture pain [29]. This analgesic effect was also apparent in OA studies in which OA patients experienced an improvement in pain after one year of intranasal sCT [30]. With calcitonin’s evident antiresorptive effects, it can prevent subchondral bone remodeling and thereby improving structural outcomes in OA [31]. However, in two phase III clinical trials, an oral formulation of sCT failed to preserve the structure, even though one of the trials showed promising relief in pain but not clinically significant, which calls for more potent stable DACRAs like KBPs [32]. An earlier study of the short-acting KBP-021 on lean OA rat model has demonstrated its effectiveness in lowering pain-like symptoms and providing structural benefits [33]. Combining the previously mentioned positive metabolic effects with the encouraging effects on OA pain and bone remodeling, KBPs have a fair chance of being a DMOAD, especially for OA patients with obesity.

In this study, we aimed to test the long-acting KBP-336 on a metabolic rat model of osteoarthritis. A high-fat diet (HFD) provided the metabolic element, while medial meniscectomy surgery (MNX) triggered OA progression. We evaluated the pain-like symptoms through the von Frey test, and by examining histological sections, we checked cartilage structure. Furthermore, we delineated which receptor mediates DACRA’s analgesic effects using selective long-acting human calcitonin.

## Methods

### Animals

Animal studies were done on Sprague Dawley (SD) rats (Taconic, Denmark) under the license (2020-15-0201-00503) issued to Nordic Bioscience by the Animal Welfare Division of the Danish Ministry of Justice. Animals were fed an ad libitum high-fat diet (HFD) containing 60% fat, 20% carbohydrates, and 20% proteins of total calories (D12492, OpenSourceDiets, Denmark) and water. Rats were housed on a 12-h light-dark cycle at 21–23 °C and 55–65% relative humidity at the animal facility of Nordic Bioscience in standard type IV cages enriched with standard wood chips, nesting material, wooden sticks, and red-tinted huts. At the study’s end, animals were euthanized by either isoflurane anesthesia followed by exsanguination or a percussive blow to the head followed by exsanguination if they weighed < 500 g.

### OA induction

A medial meniscectomy (MNX) surgery was done as previously described [33, 34]. Rats were generally anesthetized with isoflurane. After shaving the right knee, intradermal bupivacaine was injected into the knee to induce local anesthesia. Following disinfection with chlorhexidine, the medial collateral ligament is dissected, and the medial meniscus is clipped from both ends and removed. Sham animals underwent the same steps, but nothing was done to any joint structure. Postoperatively, for three days, Carprofen 5 mg/kg q24h was administered subcutaneously for postsurgical pain.

### Acute and chronic study designs

The first study was done using twenty-eight female SD rats. Animals were on HFD for 22 weeks; at 26 weeks of age, after taking baseline for the von Frey test, they were randomized according to their 50% paw withdrawal threshold (PWT) and body weight into three groups (MNX + KBP-336 4.5 nmol/kg/72h, *n* = 10), (MNX + Vehicle/72h, *n* = 10), and (Sham + Vehicle/72h, *n* = 8). KBP-336 (Bachem, Switzerland) was administered subcutaneously; treatment started three days before MNX surgery, KBP-336 dose was decided based on previous studies tested on rat models [35–37]. After eight weeks, the study was terminated; ipsilateral knee joints and adipose tissues were collected. The second study involved 36 male SD rats aged 7–8 weeks. After 12 weeks on HFD, rats were baselined and randomized according to their PWT and body weight into three groups (MNX + KBP-336 4.5 nmol/kg/72h, *n* = 14), (MNX + Vehicle/72h, *n* = 12), and (Sham + Vehicle/72h, *n* = 10. The study ended after eight weeks. The last chronic study was done using 72 female SD rats 8–9 weeks old. After 12 weeks on HFD, they were baselined and randomized according to PWT and body weight into six groups which included Vehicle, KBP-336 and human acylated calcitonin (hCTA) (SynPeptide, Shanghai, China): (MNX + hCTA 100 nmol/kg/24h, *n* = 12) (MNX + KBP-336 0.5 nmol/kg/72h, *n* = 12), (MNX + KBP-336 1.5 nmol/kg/72h, *n* = 12), (MNX + KBP-336 4.5 nmol/kg/72h, *n* = 12), (MNX + Vehicle/72h, *n* = 12), and (Sham + Vehicle/72h, *n* = 12). The study was terminated after six weeks; ipsilateral knees and adipose tissues were collected and weighed. Body weights were measured for all studies according to their dosing schedules.

Acute studies were conducted in male Sprague Dawley rats fed a standard chow diet (Altromin 1328 F Hybridpellets, Brogaarden, Denmark) containing 65% carbohydrates, 24% proteins, and 11% fat of total calories. At 12 weeks of age, prior to the test rats were allocated in treatment groups based on body weight (*n* = 8 rats per group for all groups except hCTA 250 nmol/kg group *n =* 7 in the food intake study while in the C-terminal telopeptide I (CTX-I) inhibition study, vehicle *n* = 8, hCTA 31 nmol/kg, 62 nmol/kg, 125 nmol/kg and 250 nmol/kg *n =* 4, *n =* 7, *n =* 7, and *n =* 3 rats per group, respectively). In all acute studies, overnight fasted rats received a single s.c. injection of increasing doses of hCTA or vehicle at time 0, and food intake and body weight were measured 6-, 24-, 48-, and 72 h post-injection. Blood samples were collected 6-, 24-, 48-, and 72 h post-injection to measure serum CTX-I. All acute studies were performed as cross-over studies. Serum samples were kept at -20^o^C until analysis was performed.

### Mechanical allodynia evaluation

Nociception was evaluated using the up and down method with von Frey filaments [38]. The 50% paw withdrawal threshold (PWT) of the right paw (ipsilateral paw) was used as a measure for mechanical allodynia. Rats were placed in transparent enclosures on a metal meshwork for 15–20 min to acclimatize before testing; then, they were tested with forces varying according to their responses. If a reaction occurs, the next filament used has a higher force. If no response is observed, then a lower pressure is applied. Animals were tested every nine days, 24 h after dosing if the dosing schedule was q72h, and 1–2 h after dosing for q24h dosing regimens. Tests were done in groups that included a random number of animals from each treatment group by a blinded experimenter.

### CTX-I enzyme immunoassay (EIA) and beta-arrestin recruitment assays

CTX-I assays were done according to the manufacturer’s instructions (RatLaps™ (CTX-I) EIA, Immunodiagnostic Systems, UK). Ligand selectivity was evaluated using beta-arrestin assay on the U20S cell line for the calcitonin receptor (93-0566C3 DiscoverX) and CHO K1 CALCR RAMP3 for the amylin-3 receptor (93-0268C2, DiscoverX). For more details regarding the assays refer to the supplementary material.

### Histological examination

At study termination, after terminating animals, the right ipsilateral knee was excised and stored in 4% formaldehyde for three days. The decalcification process was done using a 15% EDTA buffer for three months at room temperature. After decalcification, paraffin embedding was done using (Sakura Finetek, Alphen aan den Rijn, The Netherlands). 5 μm thick coronal sections of paraffin blocks were cut using an HM 360 microtome (Microm International GmbH, Walldorf, DE). Staining with toluidine (Merch KGaA, Darmstadt, Germany) blue was done after deparaffinization and rehydration of the sections [39]. The OA score was assessed using the Osteoarthritis Research Society International (OARSI) osteoarthritis pathology assessment system [40]. (OA score = grade x stage) The grade is the depth of cartilage damage, and the stage is how spread out is the damaged area. Blinded scoring involved the cartilage of the medial tibial plateau.

### Statistical analysis

Statistical tests and plots were done using GraphPad Prism 9 (La Jolla, CA, USA) with a significance level set at a two-tailed *P* < 0.05. Plots and mentioned values are the mean values ± standard error of the mean (SEM). Von Frey tests are represented as percentage change of the 50% paw withdrawal threshold (PWT) from baseline values. Unblinding and data analysis was done at study end. Body weight data presented are percentage changes from body weight at baseline. Two-way ANOVA was used to analyze von Frey tests for each timepoint throughout the studies. One-way ANOVA was used to analyze the net area under the curve (Net AUC) of von Frey tests, study end body weight and adipose tissues with Dunnett’s adjustment for multiple comparisons.

## Results

### KBP-336 reduces pain-like symptoms in female rats with OA

**Fig. 1:**
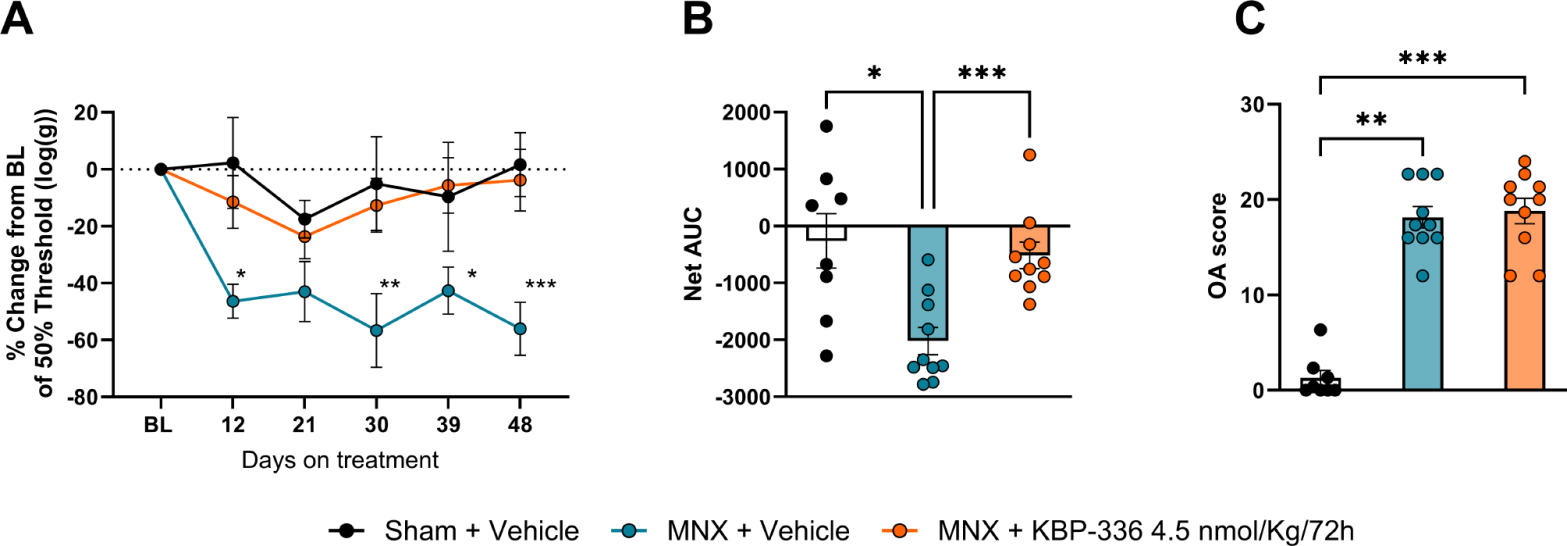
KBP-336 decreases mechanical allodynia in the metabolic OA rat model. (**A**) The mean percentage change of 50% paw withdrawal threshold (PWT) from baseline PWT of female SD rats on a high-fat diet that received sham surgery on vehicle treatment (Sham + Vehicle), meniscectomy on vehicle treatment (MNX + Vehicle), or meniscectomy on KBP-336 treatment (MNX + KBP-336 4.5 nmol/Kg/72 h) groups *n* = 8, 10, and 10 rats per group, respectively. (**B**) The net area under the curve (AUC) derived from plot (**A**). (**C**) The mean OA score for the medial tibial cartilage. Error bars indicate the standard error of the mean. Two-way ANOVA with Dunnett’s adjustment for multiple comparisons was used for (**A**). One-way ANOVA test with Dunnett’s adjustment for multiple comparisons was used for (**B**). Kruskal-Wallis test with Dunn’s adjustment for multiple comparison was used for (**C**). Statistical significance is indicated by *P < 0.05, **P < 0.01, and ***P < 0.001 vs. MNX + Vehicle (**A**)

Obese female rats on HFD were used for testing KBP-336 as a treatment for OA. We evaluated its effect on OA symptomatically and structurally. The vehicle-treated MNX group showed lower PWT in general than the sham group. KBP-336-treated rats exhibited higher PWT than the vehicle-treated MNX group, showing most sensitization on day 21, reaching − 23.6% ± 7.7. On day 48, in the last von Frey time point, the MNX + Vehicle group had a significantly lower 50% PWT than baseline − 56% ± 9.3. In contrast, the KBP-336-treated group experienced less profound pain-like symptoms − 3.8% ± 10.8 (*P* < 0.01) (Fig. 1A). Net AUC of KBP-336-treated MNX groups was substantially less negative than the vehicle-treated MNX group (*P* < 0.001) and, at the same time, not significantly different from the sham group (Fig. 1B). On the other hand, OA scores calculated through histological examination of the ipsilateral knee joint shows the extent of damage in terms of cartilage loss and bone malformations inflicted to the medial side of the knee joint. The MNX groups exhibited higher scores than the sham surgery group. The OA score of the KBP-336-treated group was not different from the vehicle-treated MNX group (Fig. 1C, Supplementary Fig. 2).

### KBP-336 decreases body weight and body fat in female rats

**Fig. 2:**
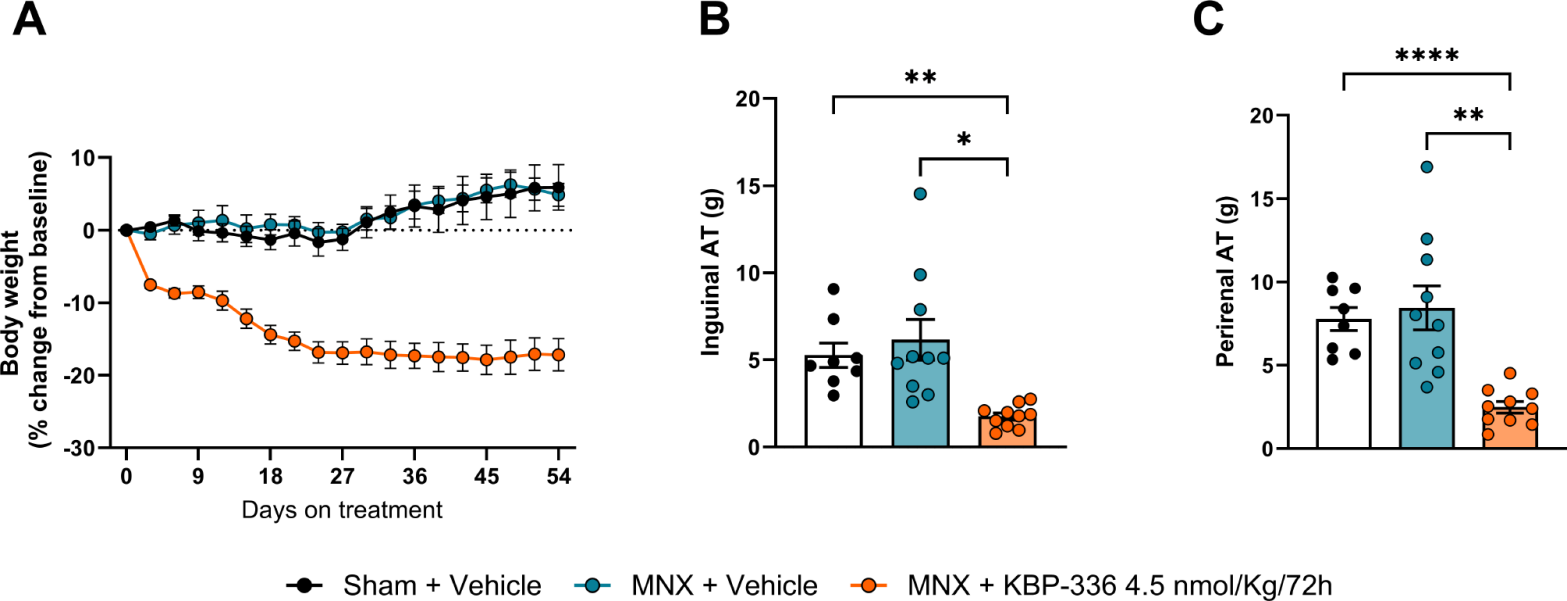
KBP-336 lowers body weight and fat depots. (**A**) The mean percentage change of body weights of female SD rats on a high-fat diet that received sham surgery on vehicle treatment (Sham + Vehicle), meniscectomy on vehicle treatment (MNX + Vehicle), or meniscectomy on KBP-336 treatment (MNX + KBP-336 4.5 nmol/kg/72 h) groups *n* = 8, 10, and 10 rats per group, respectively. (**B**) The mean weight of inguinal adipose tissue at the end of the study. (**C**) The mean weight of perirenal adipose tissue at the end of the study. Error bars indicate the standard error of the mean. One-way ANOVA test with Dunnett’s adjustment for multiple comparisons was used for analysis. Statistical significance is shown by *P < 0.05, **P < 0.01, ***P < 0.001, and ****P < 0.0001

Before beginning treatment, their BW was 377 g ± 0.4 at baseline. After 54 days of treatment, KBP-336-treated rats showed significant weight loss of 17.2% ± 2.2 compared to the vehicle-treated groups, which started to gain more weight by the end of the study (*P* < 0.0001) (Fig. 2A). Additionally, the fat depots followed the same pattern of body weight loss. The KBP-336-treated group had significantly less inguinal adipose tissue than the vehicle-treated MNX group (*P* < 0.05) (Fig. 2B). The same goes for the perirenal adipose tissues; KBP-336 reduced their masses considerably compared to the vehicle on MNX rats (*P* < 0.01) (Fig. 2C), as previously shown in earlier studies on preclinical obese models [25]. KBP-336 had a positive effect on PWT of MNX rats compared to the vehicle, in line with its effect on body weight.

### KBP-336 reduces body weight and pain-like symptoms in male rats with OA

**Fig. 3:**
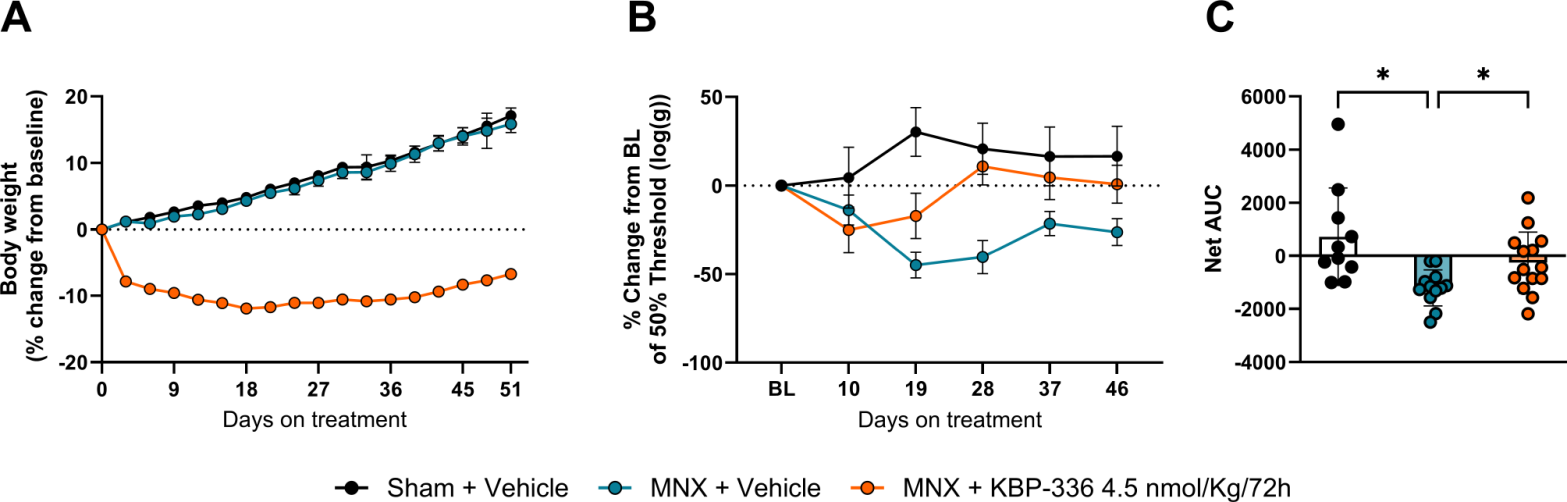
KBP-336 decreases body weight and mechanical allodynia in metabolic OA in male rats. (**A**) The mean percentage change of body weights of male SD rats on a high-fat diet that received sham surgery on vehicle treatment (Sham + Vehicle), meniscectomy on vehicle treatment (MNX + Vehicle), or meniscectomy on KBP-336 treatment (MNX + KBP-336 4.5 nmol/Kg/72 h) groups *n* = 10, 12, and 14, rats per group, respectively. (**B**) The mean percentage change of 50% paw withdrawal threshold (PWT) from baseline PWT. (**C**) The net area under the curve (AUC) derived from plot (**B**). One-way ANOVA test with Dunnett’s adjustment for multiple comparisons was used for (**A**, **C**). Error bars indicate the standard error of the mean. Statistical significance is indicated by *P < 0.05, and **** *P* < 0.0001 vs. MNX + Vehicle

In male rats, we aimed to investigate whether sex differences impact KBP-336’s effect on pain. After 51 days of treatment, the mean body weight of KBP-336-treated male rats was 498 g ± 11.6, while the vehicle-treated MNX group weighed 623 g ± 21. Vehicle-treated MNX rats were 25% heavier than their KBP-336 counterparts, showing significant KBP-336-induced weight loss (*P* < 0.0001) (Fig. 3A). Ten days post-surgery, MNX groups were slightly sensitized. From day 19 onwards vehicle-treated MNX group had lower PWT than the KBP-336 group (Fig. 3B). The derived net AUC demonstrated that Vehicle-treated MNX rats had significantly lower net AUC than KBP-336-treated MNX rats and sham rats (*P* < 0.05) (Fig. 3C).

### Human acylated calcitonin selectively activates the calcitonin receptor

**Fig. 4:**
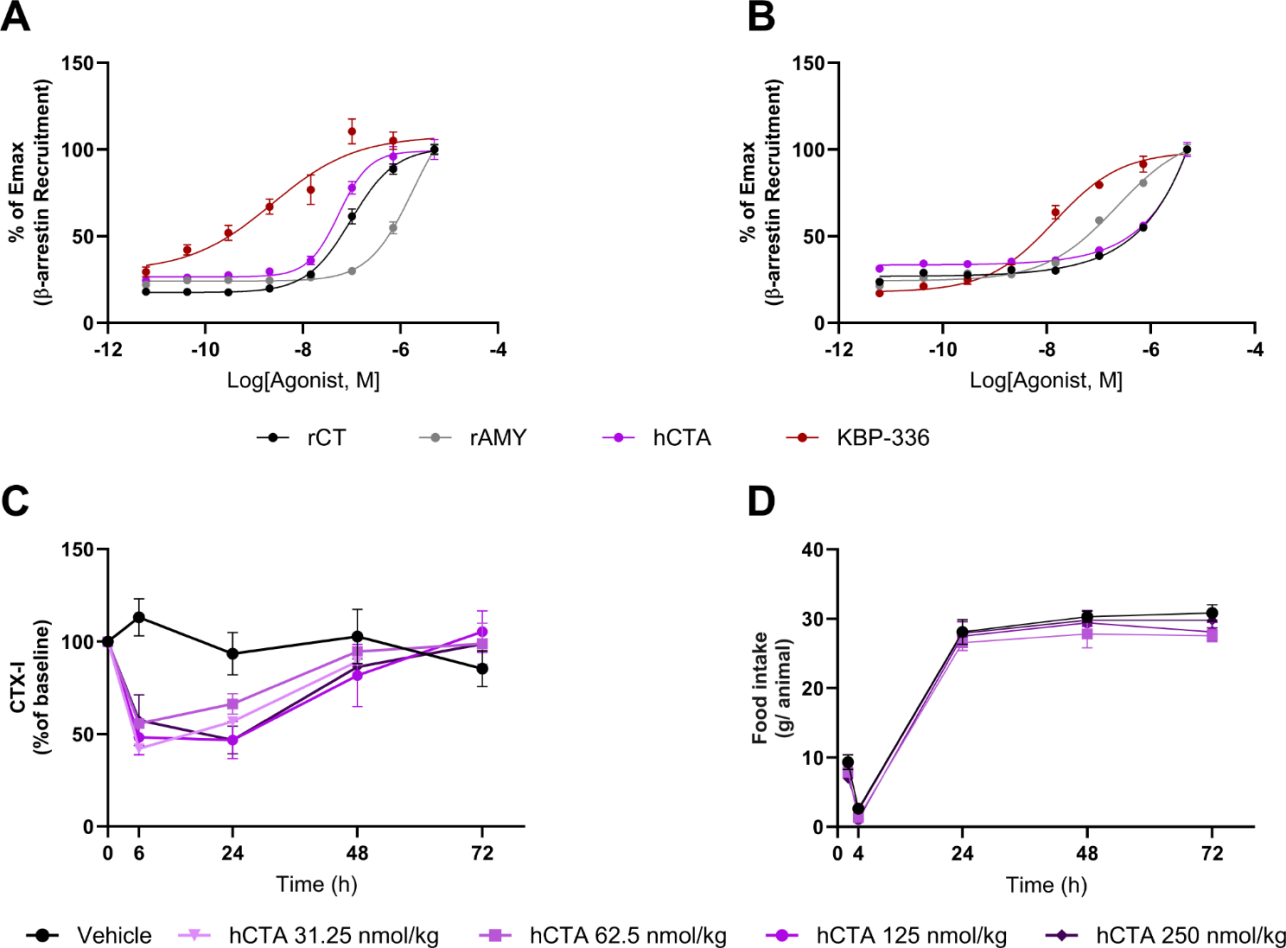
The selectivity of presumed long-acting calcitonin hCTA. (**A**) Dose-range curves of rat calcitonin (rCT), rat amylin (rAMY), long-acting human calcitonin (hCTA), and KBP-336 on induction of β-arrestin in calcitonin receptor (CTR). (**B**) Amylin receptor type 3 (AMY3-R) using *n* = 6 per data point. (**B**) The mean percentage of C-terminal telopeptide I (CTX-I) in lean SD rats after a single injection with the vehicle, hCTA at 31 nmol/kg, 62 nmol/kg, 125 nmol/kg, or 250 nmol/kg, *n* = 8, 4, 7, 7, and 3 rats per group, respectively. (**C**) The mean food intake per animal after injection with the vehicle, hCTA at 62 nmol/kg, 125 nmol/kg, or 250 nmol/kg, *n* = 8, rats per group, except for 250 nmol/kg, *n* = 7. Error bars indicate the standard error of the mean

KBP-336 is a potent DACRA. But revealing which receptor impacts pain alleviation more is a complicated matter due to the complex relationship between the amylin and calcitonin receptors [41]. To elucidate the receptor involved in the analgesic effects, we assessed the selectivity of a putative selective long-acting calcitonin receptor agonist. First, we investigated the selectivity of hCTA using the β-arrestin recruitment assay. Dose-response curves on CTR and AMY3-R expressing cells were used to examine hCTA’s activity. On CTR, hCTA matched rat calcitonin (rCT), showing more potency than rat amylin (rAMY). In contrast, rAMY bested both hCTA and rCT on AMY3-R (Fig. 4A and B). Following in vitro characterization, we checked for calcitonin receptor activity in vivo by quantifying the reduction of serum cross-linked C-telopeptide of type I collagen (CTX-I). hCTA reduced CTX-I, indicating the activation of the calcitonin receptor. hCTA maintained a profound inhibition of CTX-I up to 24 h post-dosing and wearing off after 48 h, while long-acting KBP-336 maintained CTX-I inhibition up to 48 h (Fig. 4C, Supplementary Fig. 3A). Through monitoring food intake, we can detect amylin receptor activation *in vivo.* An acute study measuring food intake indicated that hCTA did not affect food intake at any dose tested (Fig. 4D). hCTA exhibits the pharmacological profile of a CTR agonist with little to no activity on AMY3 receptor.

### KBP-336 and hCTA decrease mechanical allodynia in the metabolic OA model

**Fig. 5:**
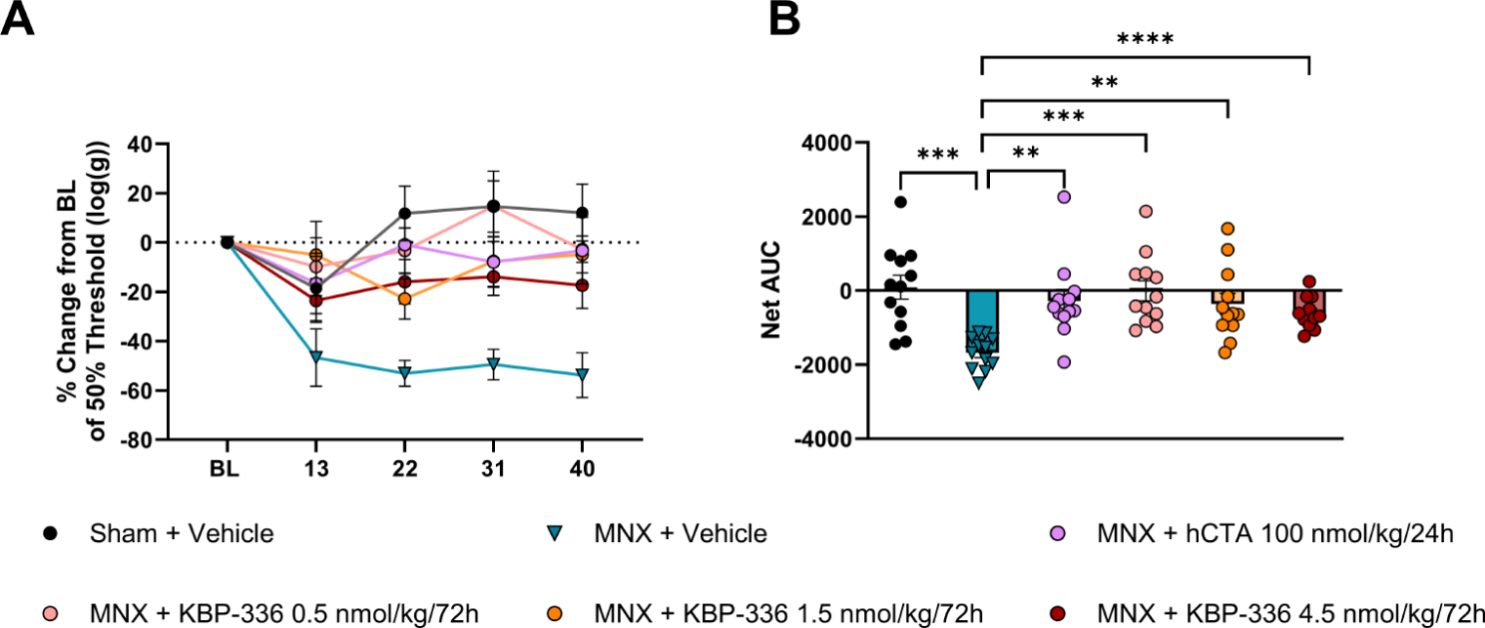
hCTA and KBP-336 at three dosing regimens decreased mechanical allodynia in the metabolic OA model in rats. (**A**) The mean percentage change of 50% paw withdrawal threshold (PWT) from baseline PWT of female SD rats on a high-fat diet that received sham surgery on vehicle treatment (Sham + Vehicle), meniscectomy on vehicle treatment (MNX + Vehicle), meniscectomy on hCTA treatment (MNX + hCTA 100 nmol/Kg/24 h), meniscectomy on KBP-336 treatment (MNX + KBP-336 0.5 nmol/Kg/72 h), meniscectomy on KBP-336 treatment (MNX + KBP-336 1.5 nmol/Kg/72 h), or meniscectomy on KBP-336 treatment (MNX + KBP-336 4.5 nmol/Kg/72 h) groups *n* = 12 rats per group. The table compares the last von Frey time point for all groups with the MNX + Vehicle group. (**B**) The net area under the curve (AUC) derived from plot (**A**). Error bars indicate the standard error of the mean. One-way ANOVA test with Dunnett’s adjustment for multiple comparisons was used for (**B**). Statistical significance is indicated by *P < 0.05, **P < 0.01, ***P < 0.001, and **** *P* < 0.0001 vs. MNX + Vehicle

After demonstrating hCTA’s selectivity through acute in vivo studies and in vitro assays, we used hCTA to uncover the receptor responsible for pain alleviation. We included other doses of KBP-336 to find out the optimal anti-nociceptive dose. Following treatment start and OA induction, we examined the progression of the pain-like symptoms through von Frey tests. After 13 days of treatment, all groups experienced a transient decrease in PWT, and then the sham surgery group returned to their baseline levels. The sham group maintained the baseline level until the end of the study. The three KBP-336 dosing regimens started with a smaller drop in PWT post-surgery and showed a significantly higher PWT than the vehicle MNX group at the study end (Supplementary Table 1). The lowest dose of KBP-336 surprisingly tended to be the least changed among them. Remarkably, hCTA-treated rats generally had PWT close to their baselines, with higher PWT than vehicle-treated MNX animals (Fig. 5A). The mean net AUC of the sham surgery group was significantly different from the MNX + Vehicle group. The net AUC of hCTA and the KBP-336-treated groups had significantly different AUC compared to the MNX + Vehicle group and not significantly different from the sham group (Fig. 5B). The medial tibial cartilage of the MNX + Vehicle and the MNX + KBP-336 4.5 nmol/Kg groups showed comparable scores (Supplementary Fig. 4).

### Chronic treatment with hCTA does not affect body weight or fat depots

**Fig. 6:**
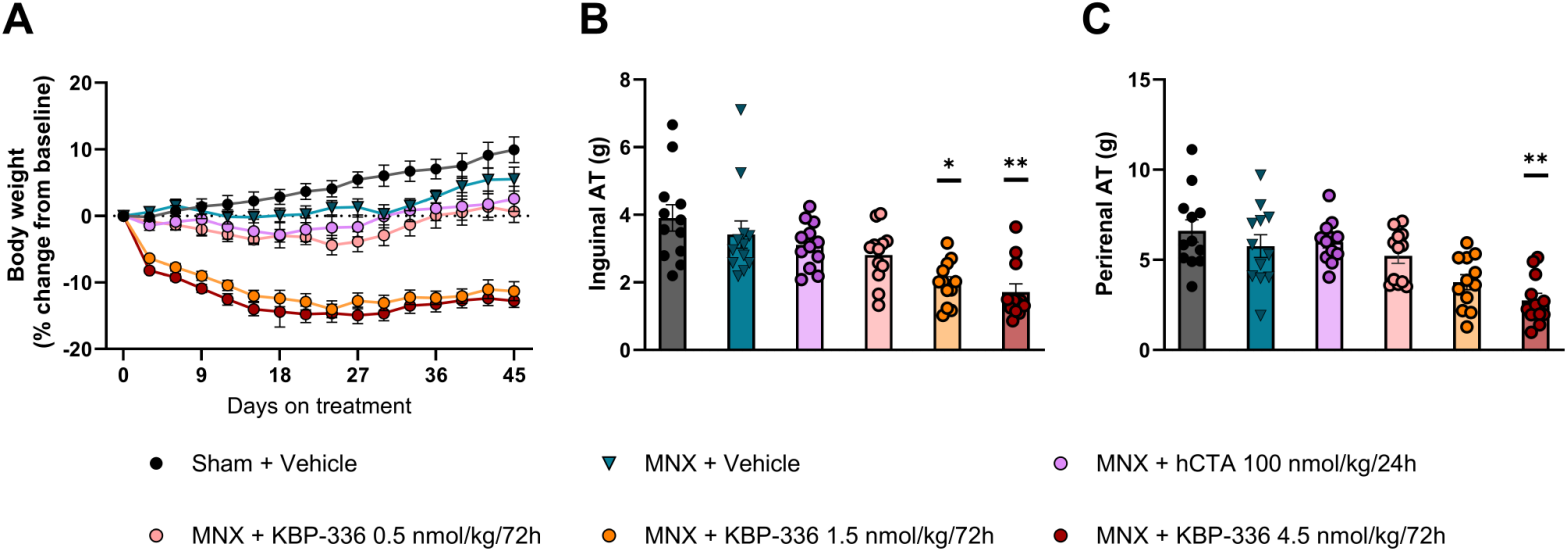
hCTA does not affect body weight and fat depots. (**A**) The mean percentage change of body weights of female SD rats on a high-fat diet that underwent sham surgery on vehicle treatment (Sham + Vehicle), meniscectomy on vehicle treatment (MNX + Vehicle), meniscectomy on hCTA treatment (MNX + hCTA 100 nmol/kg/24 h), meniscectomy on KBP-336-366 treatment (MNX + KBP-336 0.5 nmol/kg/72 h), meniscectomy on KBP-336 treatment (MNX + KBP-336 1.5 nmol/kg/72 h), or meniscectomy on KBP-336 treatment (MNX + KBP-336 4.5 nmol/kg/72 h) groups *n* = 12 rats per group. (**B**) The mean weight of inguinal and perirenal adipose tissue at the end of the study groups, *n* = 12/group. Error bars indicate the standard error of the mean. One-way ANOVA test with Dunnett’s adjustment for multiple comparisons was used. Statistical significance is shown by *P < 0.05, **P < 0.01, and **** *P* < 0.0001 vs. MNX + Vehicle

Metabolic readouts further revealed the selectivity of hCTA and the differences between KBP-336 doses. The two KBP-336 doses, 1.5 and 4.5 nmol/kg had significantly lower body weight than the vehicle-treated MNX group (*P* < 0.0001). KBP-336’s lowest dose and hCTA did not significantly reduce body weight (Fig. 6A). Fat depots reflected the observed change in body weight; KBP-336 doses of 1.5 and 4.5 nmol/kg considerably decreased inguinal adipose tissue than vehicle-treated MNX rats (*P* < 0.05, *P* < 0.01, respectively) (Fig. 6B). Only the highest dose of KBP-336 was able to significantly shrink perirenal fat compared to the MNX + vehicle group (*P* < 0.01) (Fig. 6C). The lowest dose of KBP-336 and hCTA had no statistically significant effect on all adipose tissues examined. KBP-336 0.5 nmol/kg and hCTA had a minimal impact on body weight and adiposity. hCTA and the low dose of KBP-336 had little to no effect on metabolic readouts while significantly improving mechanical allodynia.

## Discussion

Obesity fuels OA’s progression, intensifying structural damage and painful experience [42]. Of the weight loss drugs, DACRAs possess the attributes to tackle obesity and break the inflammatory cycle, as well as improve pain [33, 43]. The short-acting KBP-021 has demonstrated symptomatic and structural benefits in a lean model of OA [33]. Subsequently, we studied the effect of KBP-336, a DACRA with potent activity on calcitonin and amylin receptors, on a rat model of OA with obesity, and we shed light on the unique ability of DACRAs to provide symptom relief along with weight loss.

In meniscectomised female rats on HFD, KBP-336 treatment resulted in sustained weight loss reflected in reduced adiposity, as previously shown in obesity rat models [25, 44]. Importantly, when evaluating pain-like experience measuring mechanical allodynia through von Frey tests, rats on KBP-336 were less sensitive throughout the study. Many factors can interplay and contribute to this analgesic effect. However, cartilage structure is not among them as the KBP-336 treatment group did not show preserved or improved medial tibial cartilage. Regardless, pain-related outcomes are the main focus of regulatory authorities when evaluating DMOAD candidates [45]. In line with the study in female rats, KBP-336 treatment induced a sustained and significant weight loss in male rats. However, the treatment effect on nociception was more evident in females than in males, as we see the treatment window between the sham and MNX vehicle-treated groups narrowing towards the end of the study, unlike in the female rats. Overall, both studies confirm the previously observed DACRA-induced antinociceptive effect in a lean rat model of OA [33], thereby suggesting KBPs as potential DMOAD candidates.

The culminating pain relief can be a multitude of factors such as direct analgesic effect, decreased load on the joint, and less systemic inflammation. Our next objective was to uncover which receptor is responsible for pain alleviation. Calcitonin has been for a long time studied for its pharmacologic effects on bone and cartilage. Moreover, calcitonin has been used for bone-related pain, especially osteoporosis [29]. This analgesic effect was also apparent in OA studies as OA patients experienced an improvement in pain after one year of intranasal CT [30]. With calcitonin’s evident antiresorptive effects, it can prevent subchondral bone remodeling and thereby improve structure outcomes in OA [31]. However, most of the knowledge about calcitonin was achieved through sCT; which is a potent agonist of the amylin receptor. Through the selective long-acting calcitonin hCTA, we intended to draw clearer conclusions about which receptor had the most influence on the pain-alleviating effect. hCTA had an indistinguishable pharmacologic profile from rat calcitonin on both the calcitonin and amylin receptors. Furthermore, in vivo acute studies showed that hCTA did not affect food intake, a classical amylin-mediated response [46], while it inhibited bone resorption, hindering the release of CTX-I, consistent with calcitonin receptor activation [47], thus confirming its selectivity.

In the final animal study, we aimed to determine whether the calcitonin receptor has a direct role in pain alleviation and if the painful experience improved independently of weight loss. We also investigated an optimal dose for KBP-336 against OA pain, using lower doses that have been shown to activate the calcitonin receptor by inhibiting CTX-I levels. The effect of KBP-336 on metabolic parameters was evident, with the highest two doses inducing sizable body weight loss and smaller fat depots, while the lowest dose had a negligible effect on weight. hCTA produced a metabolic profile like the lowest dose of KBP-336; it had no significant effect on body weight and adipose tissues.

KBP-336 produced symptomatic improvement throughout the study regardless of the dose used. Although the lowest KBP-336 dose and hCTA did not affect body weight and fatty tissues, they improved pain as effectively as the higher doses of KBP-336. These findings highlight that the calcitonin receptor plays a crucial role in the anti-nociceptive effects of DACRAs, particularly KBP-336. Low doses of KBP-336 activate the calcitonin receptor, suppressing the release of CTX-I, while the amylin receptor requires higher doses to suppress food intake [22]. In line with that, hCTA and the lowest dose of KBP-336 provided analgesia through the calcitonin receptor, delivering pain-relief independent of weight. Undoubtedly, the benefits of weight loss are essential for long-term therapy of OA symptomatically and structurally. The mechanisms involved in calcitonin’s analgesic effect have been postulated before, but a definite explanation for how the calcitonin receptor activation changes pain signals is unclear [48]. Animal studies have shown that calcitonin mediates its antinociceptive effect through modulation of the activity of other neurotransmitters by altering the expression of their receptors or effector ion channels involved in signal transmission [48–50]. Regarding the effect on bone, the inhibition of CTX-I indicates a slowdown in bone remodeling, a crucial component in OA development and progression [51, 52]. And with current obesity management therapies such as GLP-1 agonist semaglutide showing increased bone resorption with no significant bone formation raising doubts about bone strength following weight loss [53]. On the other hand, the symptomatic improvement seen with the weight loss achieved through semaglutide should not be overlooked [20]. These findings highlight the importance of both weight loss combined with antiresorptive effects as it can inhibit bone remodeling while reaping the benefits of weight loss. Overall, KBP-336 provides weight loss, antinociceptive, and antiresorptive effects, targeting key components in OA pathology.

In relation to the interpretation of our results, there are some limitations to be considered. First, the MNX model combined with obesity generates a rapidly progressing disease. This rapid progression does not represent the slowly evolving nature of OA in humans and makes preserving cartilage more difficult. There was no evidence of cartilage preservation with KBP-336 treatment likely because the model is too severe, a point which is supported by previous studies showing structural protection in lean MNX rats using KBP-021 and using sCT in various models of OA decreasing cartilage damage and cartilage degradation markers, in addition to halting subchondral bone remodeling [33, 54, 55]. While the von Frey test has been shown to agree with other animal pain behavior tests [33, 56], considering an additional behavioral test can help shed more light on the treatment effects and what other symptomatic aspects it modulates. Notably, no abnormal behavior affecting food intake and pain behavior has been observed in the animals that can affect the von Frey test. It should be noted that the treatment was initiated before OA induction, so outcomes are to be interpreted in a preventive setting unlike the course of human disease development and management. Lastly, comparing KBP-336 to selective long-acting amylin can shed light on the effects of amylin on pain.

After demonstrating KBP-336’s antinociceptive and metabolic effects, a clear answer to the effects on cartilage structure is still to be revealed. A more subtly progressing model can provide the solution by closely mimicking the gradual rate of human OA development and allowing the portrayal of KBP-336’s structure-preserving capabilities.

In conclusion, metabolic benefits, pain alleviation, and potential structural preservation make DACRAs lead DMOAD candidates. KBP-336 showed anti-obesity effects in both male and female rats while significantly improving OA pain. Upon using hCTA, a selective long-acting calcitonin agonist on the metabolic OA model, the antinociceptive effects of KBP-336 proved to be mediated through the calcitonin receptor independent of weight loss or decrease in adipose tissues. KBP-336 has pain alleviating effects and can potentially be a powerful DMOAD armed with metabolic benefits against metabolically driven OA.

### Electronic supplementary material

Below is the link to the electronic supplementary material.

Supplementary Material 1

## Data Availability

No datasets were generated or analysed during the current study.

## Abbreviations

WOMAC: Western Ontario and McMaster Universities Osteoarthritis Index
DMOADs: Disease-modifying osteoarthritis drugs
HFD: High-fat diet; MNX, Meniscectomy
ND: Normal diet
OA: Osteoarthritis
PWT: Paw withdrawal thresholds
SCT: Salmon Calcitonin
DACRA: Dual Amylin and Calcitonin Receptor Agonist
hCTA: Human Acylated Calcitonin
KBP-336: KeyBioscience Peptide
