# Supplementary material for: The dual amylin and calcitonin receptor agonist KBP-336 elicits a unique combination of weight loss, antinociception and bone protection – a novel disease-modifying osteoarthritis drug
Source: Arthritis Res Ther. 2024 Jul 12;26:129. doi: 10.1186/s13075-024-03361-2 (PMC11241783; doi:10.1186/s13075-024-03361-2)
Supplement: Supplementary file 1 — Supplementary Material 1 [file 13075_2024_3361_MOESM1_ESM.docx]

# Supplementary materials

## Methods

### CTX-I enzyme immunoassay (EIA)

CTX-I assays were done according to the manufacturer’s instructions (RatLaps™ (CTX-I) EIA, Immunodiagnostic Systems, UK). Briefly, kit reagents and serum samples were allowed to thaw and reach room temperature. The biotinylated antigen of CTX-I epitope is added to the streptavidin-coated plate and incubated for 30 mins, followed by adding standards, samples, and primary rabbit antibody against the epitope after washing. After overnight incubation, a peroxidase-conjugated anti-rabbit antibody was added and incubated for 1 hour after washing. Tetramethybenzidine (TMB) substrate solution was added and incubated for 15 mins before adding the H2SO4 stop solution. The absorbance was measured at a wavelength of 450 nm with 650 nm as a reference.

Beta-arrestin recruitment assay

Ligand selectivity was evaluated using beta-arrestin assay on the U20S cell line for the calcitonin receptor (93-0566C3 DiscoverX) and CHO K1 CALCR RAMP3 for the amylin-3 receptor (93-0268C2, DiscoverX). Two thousand five hundred cells/well were seeded and incubated overnight in white 384-well plates in 10µL of each cell line’s respective medium (784080, Greiner Bio-One). After stimulation with ligands, the beta-arrestin signal from GPCR activation is detected and quantified using PathHunter Detection Kit (93- 0001, DiscoverX).

#### Supplementary data

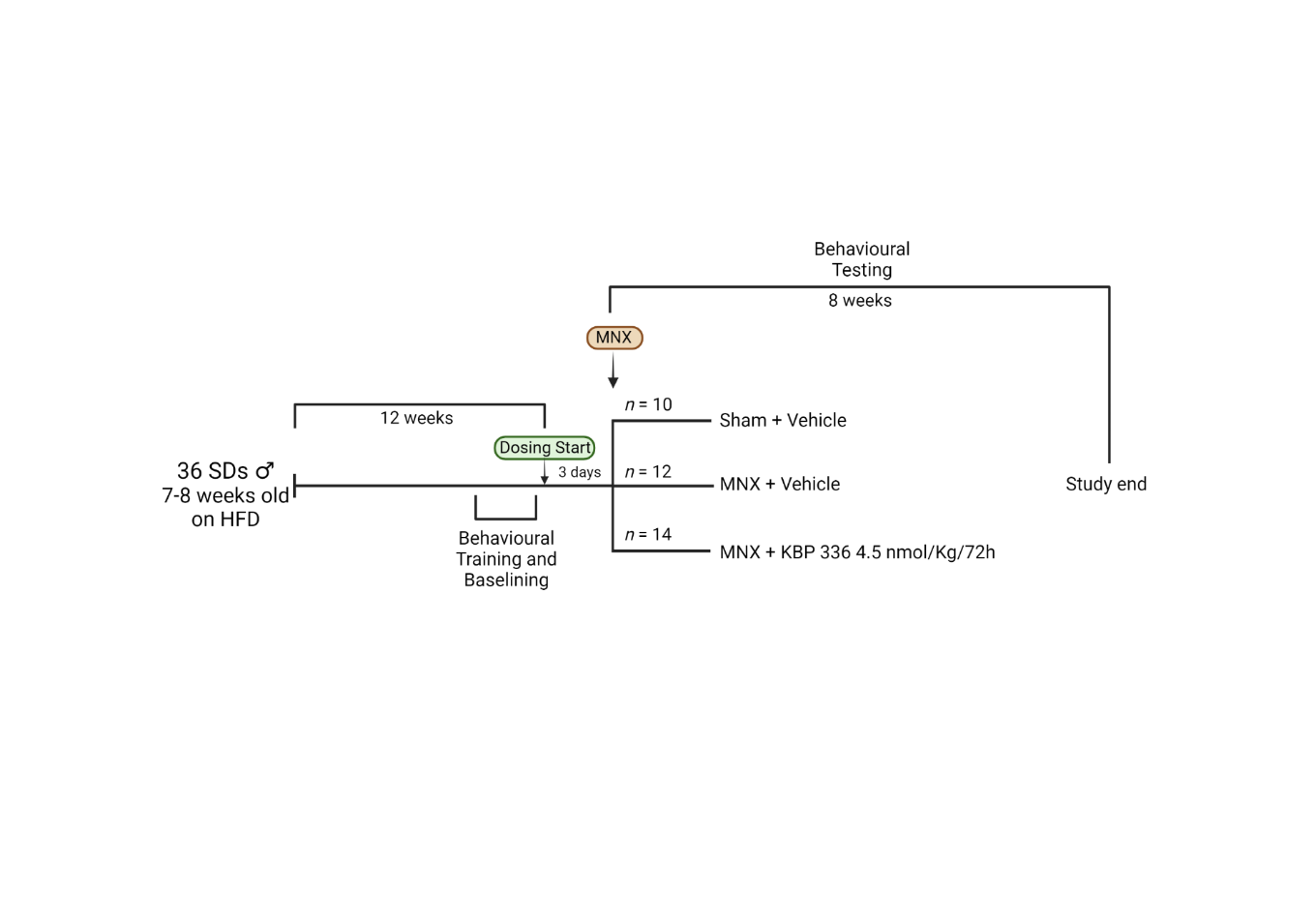


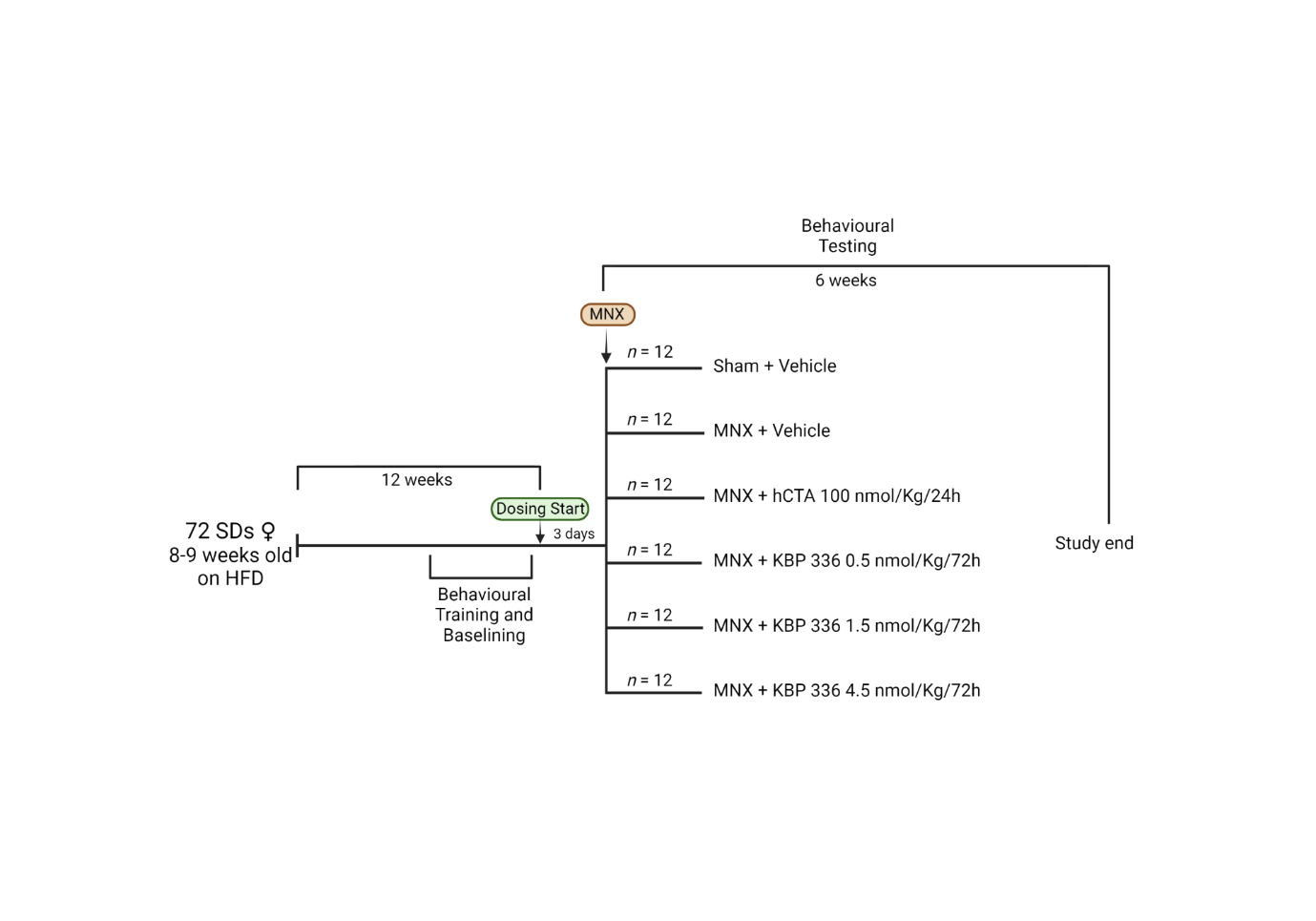


**Fig. 1** Graphical illustration depicting chronic dosing study designs, created with BioRender.com.

**Fig. 2** Representative micrographs of toluidine blue stained medial tibial cartilage sections of female SD rats on a high-fat diet that received sham surgery on vehicle treatment (Sham + Vehicle), meniscectomy on vehicle treatment (MNX + Vehicle), or meniscectomy on KBP-366 treatment (MNX + KBP-336 4.5 nmol/Kg/72h). Photos were taken at 4x magnification, and the scale bar represents 200 µm.

**Fig. 3** A) The mean percentage of C-terminal telopeptide I (CTX-I) in lean SD rats after one injection with the vehicle, KBP-336 at 0.167, 0.375, 0.5, 0.75, and 1.5 nmol/Kg, n = 8, 4, 4, 3, and 6 rats per group, respectively. B) The mean food intake per animal after one injection with the vehicle, KBP-336 at 0.167, 0.375, 0.5, 0.75, and 1.5 nmol/Kg, n = 8, 7, 7, and 7 rats per group, respectively. Error bars indicate the standard error of the mean (Previously published by Larsen et al. 2022 (46)).

B

A

**Fig. 4** A) The mean OA score for the medial tibial cartilage of female SD rats on a high-fat diet that received meniscectomy on vehicle treatment (MNX + Vehicle), or meniscectomy on KBP-366 treatment (MNX + KBP-336 4.5 nmol/Kg/72h) groups, *n* = 12 per group. Error bars indicate the standard error of the mean. B) Representative micrographs of toluidine blue stained medial tibial cartilage sections.

| Days | Two-way ANOVA comparisons | P |
| --- | --- | --- |
| 13 |  |  |
|  | MNX + Vehicle vs. MNX + KBP-336 0.5 nmol/kg/72h | * |
|  | MNX + Vehicle vs. MNX + KBP-336 1.5 nmol/kg/72h | ** |
| 22 |  |  |
|  | MNX + Vehicle vs. MNX + hCTA 100 nmol/kg/24h | *** |
|  | MNX + Vehicle vs. MNX + KBP-336 0.5 nmol/kg/72h | ** |
|  | MNX + Vehicle vs. MNX + KBP-336 4.5 nmol/kg/72h | * |
|  | MNX + Vehicle vs. Sham + Vehicle | **** |
| 31 |  |  |
|  | MNX + Vehicle vs. MNX + hCTA 100 nmol/kg/24h | * |
|  | MNX + Vehicle vs. MNX + KBP-336 0.5 nmol/kg/72h | **** |
|  | MNX + Vehicle vs. MNX + KBP-336 1.5 nmol/kg/72h | ** |
|  | MNX + Vehicle vs. MNX + KBP-336 4.5 nmol/kg/72h | * |
|  | MNX + Vehicle vs. Sham + Vehicle | **** |
| 40 |  |  |
|  | MNX + Vehicle vs. MNX + hCTA 100 nmol/kg/24h | *** |
|  | MNX + Vehicle vs. MNX + KBP-336 0.5 nmol/kg/72h | *** |
|  | MNX + Vehicle vs. MNX + KBP-336 1.5 nmol/kg/72h | ** |
|  | MNX + Vehicle vs. MNX + KBP-336 4.5 nmol/kg/72h | * |
|  | MNX + Vehicle vs. Sham + Vehicle | **** |

**Table 1** Shows a two-way ANOVA analysis with Dunnett’s adjustment for multiple comparing the mean percentage change of body weights of female SD rats on a high-fat diet that underwent sham surgery on vehicle treatment (Sham + Vehicle), meniscectomy on vehicle treatment (MNX + Vehicle), meniscectomy on hCTA treatment (MNX + hCTA 100 nmol/Kg/24h), meniscectomy on KBP-366 treatment (MNX + KBP-336 0.5 nmol/Kg/72h), meniscectomy on KBP-366 treatment (MNX + KBP-336 1.5 nmol/Kg/72h), or meniscectomy on KBP-366 treatment (MNX + KBP-336 4.5 nmol/Kg/72h) groups n = 12 rats per group. Statistical significance is shown by *P < 0.05, **P < 0.01, ***P < 0.001, and **** P < 0.0001 vs. MNX + Vehicle.
